# Large inter-arm systolic blood pressure difference is associated with cognitive impairment in older adults: a cross-sectional study in rural southwest China

**DOI:** 10.3389/fnagi.2025.1489033

**Published:** 2025-04-14

**Authors:** Qingyue Wu, Jingyuan Yang, Xunqiong Zhou, Mingyan Chen, Xing Yang

**Affiliations:** ^1^The Key Laboratory of Environmental Pollution Monitoring and Disease Control, Ministry of Education, School of Public Health, Guizhou Medical University, Guiyang, China; ^2^Xingyi People’s Hospital, Xingyi, China; ^3^School of Medical and Health Management, Guizhou Medical University, Guiyang, China

**Keywords:** cognitive impairment, inter-arm blood pressure difference, elderly, rural areas, cross-sectional study

## Abstract

**Background:**

Studies have shown that both inter-arm blood pressure difference (IABPD) and cognitive impairment are associated with vascular events. However, the relationship between IABPD and cognitive impairment among elderly individuals in rural China remains unclear. This study aims to investigate the association between IABPD and cognitive impairment in rural older adults in Guizhou, southwestern China.

**Methods:**

The study data were obtained from the Cohort Study of the Health Status of Guizhou Rural Older Adults in China (SHGROC). A multi-stage cluster sampling method was employed to select 1,088 rural elderly individuals aged ≥ 60 years from Guizhou Province for questionnaire surveys, physical examinations, and biological sample collection. Cognitive function of participants was assessed using the Mini-Mental State Examination (MMSE). Bilateral blood pressure was measured simultaneously using an automated device, and the IABPD was calculated. Multivariable linear and logistic regression models were used to examine the relationship between IABPD and cognitive impairment.

**Results:**

The overall prevalence of cognitive impairment in the study sample was 27.85%, and it was more common among participants with an IABPD ≥ 10 mmHg (*P* < 0.05). Multivariable regression analysis revealed that an inter-arm systolic blood pressure difference (IASBPD) ≥ 10 mmHg was independently associated with lower MMSE scores (β = −1.113; 95% *CI*: −2.120, −0.106; *P* = 0.030) and a higher risk of cognitive impairment (*OR* = 1.902; 95% *CI*: 1.189, 3.040; *P* = 0.007). Additionally, a dose-response relationship was observed between IASBPD and the risk of cognitive impairment, with a linear positive correlation. Further subgroup analysis indicated that the relationship between IASBPD and cognitive impairment was modified by sex, smoking, and regular exercise (*P* for interaction < 0.05).

**Conclusion:**

IASBPD ≥ 10 mmHg is associated with an increased risk of cognitive impairment in rural Chinese older adults. This suggested that IASBPD may provide a reference for early identification of individuals at risk of cognitive impairment.

## 1 Introduction

Cognitive impairment broadly refers to various degrees of cognitive impairment from various causes, ranging from mild cognitive impairment (MCI) to dementia (Xue et al., 2022). With the progression of global aging, age-related cognitive impairment has become a growing global public health problem (Weuve et al., 2014). Dementia, the most severe stage of cognitive impairment, is the leading cause of disability among individuals aged 60 and above worldwide (World Health Organization, 2020). It has been estimated that there were more than 50 million people with dementia worldwide in 2019, and this number is expected to increase to 152 million by 2050 (Alzheimer’s Association, 2019). Since there are still no effective treatments for dementia, early identification of individuals at risk for cognitive impairment is crucial for its prevention and management.

Growing evidence suggests a strong association between vascular health and cognitive function (Shen et al., 2020). The latest Lancet standing Commission reported that vascular lesions are not only linked to all-cause dementia but may also play an additive or interactive role in inducing cognitive impairment (Livingston et al., 2024). Additionally, cardiovascular risk factors such as arterial stiffness, hypertension, and diabetes can promote the onset and progression of cognitive impairment by accelerating vascular aging and inducing cerebral microvascular damage (Mok et al., 2024). Among various vascular health indicators, blood pressure levels are considered a significant factor influencing cognitive function. Inter-arm blood pressure difference (IABPD), as a physiological indicator that is inextricably linked to blood pressure, has gradually attracted the attention of the medical community in recent years (Clark et al., 2014). IABPD is a difference in blood pressure between the right and left arms due to the anatomical structure of the human body and many pathological factors, and is commonly used in clinical practice to evaluate atherosclerosis and poor vascular function (Essa and Ahmed, 2022). Previous studies have shown that the detection rates of IABPD ≥ 10 mmHg are 12.8% in hypertensive patients and 9.8% in diabetic patients, significantly higher than the 3.0% observed in the general population (Yu Y. et al., 2021; Lee et al., 2020). Additionally, individuals with IABPD ≥ 10 mmHg have a 2.96-fold increased risk of cardiovascular events and a 1.63-fold increased risk of all-cause mortality compared to those with IABPD < 10 mmHg (Clark et al., 2016). Notably, cardiovascular risk factors have been strongly associated with increased risk for future cognitive impairment in healthy individuals (Livingston et al., 2024).

Given the close relationship between IABPD and cardiovascular health, some academics have put out the scientific theory that there is a connection between IABPD and cognitive function. A cohort study conducted on Italian older adults aged 65 years and older found a possible association between IABPD ≥ 5 mmHg and cognitive decline (Clark et al., 2020). Another study based on the Framingham Heart Cohort indicated that high IABPD was associated with an increased risk of dementia events in older subjects carrying the APOE ε4 allele (Pase et al., 2016). Although the exact mechanism remains unknown, higher IABPD may cause vascular endothelial injury and thus increase cerebrovascular permeability and blood–brain barrier leakage, exacerbating the risk of cognitive dysfunction (Hughes et al., 2016). In addition, IABPD is closely related to hypertension, atherosclerosis, and cerebral small vessel disease. And all of these pathological changes have also been shown to be strongly associated with cognitive impairment (Livingston et al., 2024; Clark et al., 2016; Lee et al., 2022). These findings suggest that IABPD may be of potential value in cognitive screening.

Unfortunately, current research on the relationship between IABPD and cognitive impairment remains limited and primarily focused on Western developed countries, leaving it uncertain whether these findings are applicable to developing nations (Clark et al., 2020; Pase et al., 2016). Moreover, existing studies predominantly concentrate on the association between inter-arm systolic blood pressure difference (IASBPD) and cognitive function, without adequately considering the role of inter-arm diastolic blood pressure difference (IADBPD). This may lead to a one-sided understanding of their complex relationship. Additionally, the association between IABPD and cognitive impairment may be influenced by confounding variables such as demographic characteristics, lifestyle factors, and cardiovascular risk factors. Therefore, future research should include populations of diverse ethnicities and economic backgrounds, consider both IASBPD and IADBPD, and adjust for potential confounding variables to provide a more comprehensive understanding of the relationship between IABPD and cognitive impairment.

As the developing country with the largest elderly population in the world, China faces a high prevalence of dementia, particularly in rural areas (Chen et al., 2022). Studies have found that rural regions in China experience a deeper level of aging (23.81 vs. 15.82%) and a higher prevalence of cognitive impairment (48.53 vs. 36.62%) compared to urban areas (National Bureau of Statistics of China, 2021; Wang et al., 2022). Therefore, we conducted a population-based cross-sectional study in rural areas of Guizhou Province in southwestern China to explore the relationship between IABPD and cognitive impairment among the elderly. This study aims to provide scientific evidence for the early identification and management of individuals at high risk of cognitive impairment.

## 2 Materials and methods

### 2.1 Study population and design

**FIGURE 1:**
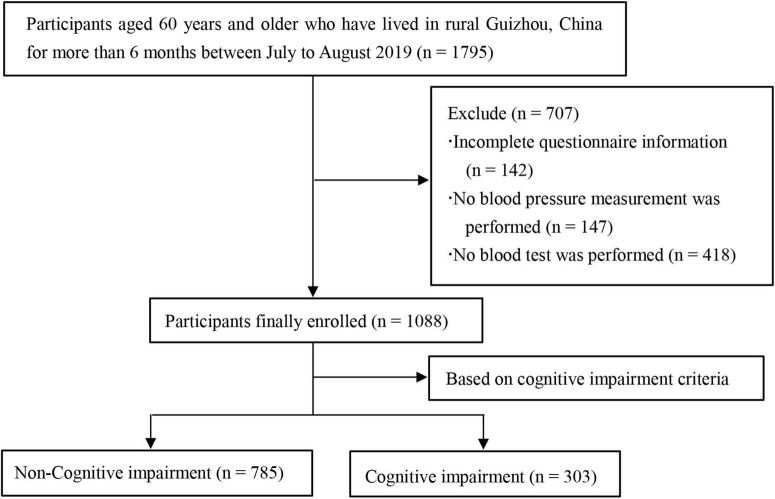
Flowchart showing the procedure used for participants recruitment.

We conducted a cross-sectional study and the data comes from baseline survey of the cohort study on the health status of Guizhou rural older adults in China (SHGROC) (Hu et al., 2022). The SHGROC is a population-based prospective study conducted in rural areas of Guizhou, southwestern China. From July to August 2019, a multistage cluster sampling method was used to select rural older adults aged ≥ 60 years from 12 villages in 2 counties (districts) of Guizhou Province, and baseline survey carried out among them. The exact sampling process is shown in Supplementary Figure 1. The participant inclusion criteria were: (1) those who were older adults of 60 years and above; (2) those who had lived in their current residence for more than 6 months. Exclusion criteria were: (1) those who suffer from severe visual and hearing impairment, physical disability, aphasia or other reasons that prevent them from cooperating with the examination; (2) those who have been diagnosed with dementia or other mental illness. A total of 1,795 questionnaires were distributed, and data from 1,088 participants were finally included in the study by excluding subjects with incomplete information on the questionnaires, those who did not undergo blood pressure measurements, and those who did not undergo blood tests (Figure 1). All participants signed the informed consent form and the study was approved by the medical ethics committee of Guizhou Medical University (approval No. 2017-049).

### 2.2 Data collection methods

This study conducted an on-site, centralized survey of participants on a face-to-face, one-on-one basis. Questionnaires, anthropometric measurements and biological samples were collected by trained investigators. We collected information regarding cognitive function, demographic characteristics, lifestyle behaviors, and chronic disease history using a standardized questionnaire. Data on blood pressure, height, and weight were collected through physical examinations. Fasting venous blood samples were obtained and analyzed by professional technicians.

### 2.3 Cognitive assessment

The Mini-Mental State Examination (MMSE) was used to assess the cognitive function of the participants (Folstein et al., 1975). The MMSE is an internationally recognized cognitive screening tool that has demonstrated good test–retest reliability and concurrent, criterion, and construct validity, and is more applicable to questionnaires for rural older adults (Shigemori et al., 2010). In this study, our data analysis demonstrated good internal consistency of the MMSE scale (Cronbach’s α = 0.876) among the studied population. The questionnaire consists of 11 main items assessing abilities in five domains: orientation, immediate memory, attention and calculation, language, and delayed recall. The total MMSE score ranges from 0 to 30 points, with lower scores reflecting worse cognitive function (Creavin et al., 2016). As previously reported, we used a cutoff MMSE score below 18 points to define cognitive impairment (Zhang et al., 2008; Yu Y. et al., 2021; Mao et al., 2020).

### 2.4 IABPD measurement

Brachial blood pressure was measured simultaneously using the Atherosclerosis detector HBP-8000 (Omron, Japan). The measurements obtained by this device are highly consistent with standard methods, demonstrating excellent repeatability and reliability, and it has been widely used in previous studies (Shang et al., 2024; Qu et al., 2022). The temperature of the examination room was kept at 22∼25°C. On the day of the test, subjects should avoid strenuous exercise, smoking, drinking alcohol or caffeine-containing beverages, and sit still for at least 15 min before taking the test. During the measurement, subjects were asked to lie in a supine position, keep quiet, and place both hands palm up on both sides of the body. Trained technicians placed pressure cuffs on both arms and measured blood pressure in both arms at the same time. Measurements were repeated twice and averaged. IABPD was calculated as the absolute value of the difference in blood pressure between the right and left upper extremities (Yu H. et al., 2021). According to the National Institute for Health and Care Excellence (NICE) guidelines and previous literature (National Clinical Guideline Centre (UK), 2011; Clark et al., 2021), abnormal IABPD was defined as an absolute differences ≥ 10 mmHg, including IASBPD ≥ 10 mmHg and IADBPD ≥ 10 mmHg.

### 2.5 Definition of covariates

We selected the following covariates as potential confounders based on the relevant literature (Livingston et al., 2024; Mouseli et al., 2024): (1) Demographics: including age (< 70 or ≥ 70 years), sex (male or female), education level (≤ 6 years or > 6 years), and marital status (“married” included current marriage or partnership; “unmarried” included single, divorced, separated and widowed). (2) Behavior and lifestyle: including smoking (“yes” for current smokers; “no” for former smokers and never smokers), alcohol consumption (similarly classified as smoking), and regular exercise (yes or no). (3) Chronic diseases: including obesity, hypertension, diabetes, dyslipidemia, and cardio-cerebral vascular disease (CCVD). Obesity was defined as a body mass index (BMI) ≥ 25 kg/m^2^ according to the World Health Organization’s recommendations for classification of Asian populations (WHO Expert Consultation, 2004). Hypertension was defined as any systolic blood pressure ≥ 140 mmHg or diastolic blood pressure ≥ 90 mmHg or self-reported history of hypertension or use of antihypertensive medication (Williams et al., 2004). Diabetes mellitus was defined as fasting glucose ≥ 7.0 mmol/L or self-reported history of diabetes mellitus or use of glucose-lowering drugs (ElSayed et al., 2023). Dyslipidemia was defined if patients have one or more of the following conditions: TC ≥ 6.22 mmol/L, LDL-C ≥ 4.14 mmol/L, HDL-C ≤ 1.04 mmol/L, TG ≥ 2.26 mmol/L or self-reported history of dyslipidemia or use of anti-dyslipidemia medication (Joint committee for guideline revision, 2018).

### 2.6 Quality control

All investigators received extensive training relative to the study questionnaire and outcome measures before conducting the investigation. During the investigation, researchers selected eligible participants in accordance with the inclusion and exclusion criteria, and conducted on-site investigations using a unified protocol. After the questionnaire survey, two reviewers conducted on-site verification to ensure the completeness of the questionnaire responses. Additionally, blood pressure measurements from both arms were taken by three trained investigators using professional equipment to ensure the accuracy of the measurements.

### 2.7 Statistical analysis

Continuous, normally distributed variables were represented by mean and standard deviation (SD), continuous variables with skewed distribution by median (interquartile range), and categorical variables were represented as percent (%). The chi-square test was used to compare the prevalence of cognitive impairment in older adults with different characteristics, and the Mann-Whitney U test was used to compare MMSE scores between different IABPD groups. Multivariate linear regression and logistic regression analysis were used to evaluate the association between IABPD and cognitive impairment. Covariates were selected based on their availability and potential relationship with cognitive impairment and IABPD. All covariates that were significant (*P* < 0.05) in the univariate analysis were also included in the multivariate model. Regression analysis established four models: model 1 was not adjusted for covariates; model 2 was adjusted for demographic characteristics (age, sex, marital status, and education); model 3 was adjusted for cardiovascular risk factors (smoking, regular exercise, hypertension, and CCVD); and model 4 was adjusted for all the variables in models 2 and 3 were. Restricted cubic spline regression with five knots at the 5th, 27.5th, 50th, 72.5th, 95th percentiles was used to explore the potential dose–response relationship between IABPD and cognitive impairment. Furthermore, stratified and interaction analyses were performed to examine whether the association between IABPD and cognitive impairment differed by age, sex, marital status, education, smoking, regular exercise, hypertension, and CCVD. We used complete participant data for our analyses and did not impute missing data. All statistical analyses were performed in R software (version 4.3.3) with a two-tailed *P*-value < 0.05 as statistically significant.

## 3 Results

### 3.1 Participants characteristics

Table 1 shows the overall characteristics of all participants and a comparison of the characteristics of participants with and without cognitive impairment. A total of 1,088 participants were included in this study, including 627 (57.63%) females and 461 (42.37%) males, with a mean age of 71.2 ± 6.4 years (range: 60-96 years), 87.78% had primary education or less, and more were married (61.86%). The mean MMSE score of the participants was (20.90 ± 5.48), and a total of 303 patients with cognitive impairment were identified, with a prevalence of 27.85%. Among them, age > 70 years, female, education level ≤ 6 years, married, smoker, lack of physical activity, having hypertension, having CCVD, IASBPD ≥ 10 mmHg, and IADBPD ≥ 10 mmHg were significantly more common in patients with cognitive impairment (*P* < 0.05). In addition, 99 (9.10%) of the participants had an IASBPD ≥ 10 mm Hg and 80 (7.35%) had an IADBPD ≥ 10 mm Hg, a comparison of the prevalence of IABPD in older adults with different characteristics is shown in Supplementary Table 1.

**TABLE 1:** Comparison of cognitive impairment prevalence among rural elderly with different characteristics.

Variables	All (*n* = 1,088)	Cognitive status		χ^2^	*P*-value
		Impairment (*n* = 303)	No impairment (*n* = 785)		
**Age, years**					
<70	480 (44.12)	91 (30.03)	389 (49.55)	33.791	**<0.001**
≥70	608 (55.88)	212 (69.97)	396 (50.45)		
**Sex**					
Male	461 (42.37)	68 (22.44)	393 (50.06)	68.307	**<0.001**
Female	627 (57.63)	235 (77.56)	392 (49.94)		
**Education, years**					
≤ 6	955 (87.78)	297 (98.08)	658 (83.82)	41.072	**<0.001**
>6	133 (12.22)	6 (1.98)	127 (16.18)		
**Marital status**					
Unmarried	415 (38.14)	149 (49.17)	266 (33.89)	21.660	**<0.001**
Married	673 (61.86)	154 (50.83)	519 (66.11)		
**Smoking**					
No	791 (72.70)	256 (84.49)	535 (68.15)	29.395	**<0.001**
Yes	297 (27.30)	47 (15.51)	250 (31.85)		
**Drinking**					
No	798 (73.35)	232 (76.57)	566 (72.10)	2.230	0.135
Yes	290 (26.65)	71 (23.43)	219 (27.90)		
**Regular exercise**					
No	582 (53.49)	189 (62.38)	393 (50.06)	13.322	**<0.001**
Yes	506 (46.51)	114 (37.62)	392 (49.94)		
**BMI, kg/m^2^**					
>25	844 (77.57)	238 (78.55)	606 (77.20)	0.229	0.632
≥25	244 (22.43)	65 (21.45)	179 (22.80)		
**Hypertension**					
No	404 (37.13)	95 (31.35)	309 (39.36)	6.008	**0.014**
Yes	684 (62.87)	208 (68.65)	476 (60.64)		
**Dyslipidemia**					
No	896 (82.35)	246 (81.19)	650 (82.80)	0.392	0.531
Yes	192 (17.65)	57 (18.81)	135 (17.20)		
**Diabetes**					
No	1,019 (93.66)	281 (92.74)	738 (94.01)	0.597	0.440
Yes	69 (6.34)	22 (7.26)	47 (5.99)		
**CCVD**					
No	986 (90.62)	284 (93.73)	702 (89.43)	4.764	**0.029**
Yes	102 (9.38)	19 (6.27)	83 (10.57)		
**IASBPD**					
<10 mmHg	989 (90.90)	263 (86.80)	726 (92.48)	8.543	**0.003**
≥10 mmHg	99 (9.10)	40 (13.20)	59 (7.52)		
**IADBPD**					
<10 mmHg	1,008 (92.65)	271 (89.44)	737 (93.89)	6.345	**0.012**
≥10 mmHg	80 (7.35)	32 (10.56)	48 (6.11)		

Data are n (%). BMI, body mass index; CCVD, cardio-cerebral vascular disease. Bold values indicate statistically significant differences.

### 3.2 Association of IABPD with cognitive impairment

As shown in Table 2, those with IASBPD ≥ 10 mmHg had a higher prevalence of cognitive impairment and lower MMSE scores, compared to those with IASBPD < 10 mmHg (*P* < 0.05). Table 3 shows the multivariate linear regression with MMSE score as dependent variable and IASBPD and IADBPD as independent variables. In model1, which was not adjusted for confounders, higher level of IASBPD was associated with lower MMSE score (β = −1.408; 95% *CI*: −2.539, −0.277; *P* = 0.015). In the fully adjusted model, the association remained significant (β = −1.113;95% *CI*:−2.120, −0.106; *P* = 0.030) after adjusting for demographic characteristics (age, sex, marital status, education) and cardiovascular risk factors (regular exercise, smoking, hypertension, CCVD). Furthermore, IADBPD ≥ 10 mmHg was negatively associated with the MMSE score (Model 1: β = −2.087; 95% *CI*: −3.331, −0.843; *P* = 0.001). However, this association was not significant after adjusting for covariates (*P* > 0.05).

**TABLE 2:** Relationship between IABPD and cognitive function.

Variables	MMSE score				Cognitive impairment	
	Medians (IQRs)	*Z*-value	*P*-value	*n* (%)	*χ^2^*-value	*P*-value
**IASBPD**						
<10 mmHg	21 (17, 25)	−2.334	**0.020**	263 (26.59)	8.543	**0.003**
≥10 mmHg	20 (14, 24)			40 (40.40)		
**IADBPD**						
<10 mmHg	21 (17, 25)	−3.183	**0.001**	271 (26.88)	6.345	**0.012**
≥10 mmHg	19.5 (14, 23)			32 (40.00)		

MMSE, Mini Mental State Examination; IASBPD, inter-arm systolic blood pressure difference; IADBPD, inter-arm diastolic blood pressure difference. Bold values indicate statistically significant differences.

**TABLE 3:** Results of multivariate linear regression of IASBPD and IADBPD with MMSE scores.

	MMSE, scores, β (95%*CI*), *P*-value			
	Model 1	Model 2	Model 3	Model 4
**IASBPD**				
<10 mmHg	1	1	1	1
≥10 mmHg	−1.408 (−2.539, −0.277), **0.015**	−1.119 (−2.127, −0.110), **0.030**	−1.211 (−2.305, −0.118), **0.030**	−1.113 (−2.120, −0.106), **0.030**
**IADBPD**				
<10 mmHg	1	1	1	1
≥10 mmHg	−2.087 (−3.331, −0.843), **0.001**	−0.775 (−1.901, 0.351), 0.177	−1.482 (−2.686, −0.277), **0.016**	−0.745 (−1.864, 0.374), 0.192

Model 1: No adjustment for covariates. Model 2: Demographic characteristics (age, sex, marital status, education) were adjusted. Model 3: Cardiovascular risk factors (smoking, regular exercise, hypertension, CCVD) were adjusted. Model 4: All variables in models 2 and 3 were adjusted. The *R*^2^ of IASBPD model4 was 0.237, and the adjusted *R*^2^ was 0.231. The *R*^2^ of IADBPD model4 was 0.235, and the adjusted *R*^2^ was 0.228. β, regression coefficient; *CI*, confidence interval; MMSE, Mini Mental State Examination; IASBPD, inter-arm systolic blood pressure difference; IADBPD, inter-arm diastolic blood pressure difference; CCVD, cardio-cerebral vascular disease. Bold values indicate statistically significant differences.

Table 4 shows the results of multivariate logistic regression analysis with IASBPD and IADBPD as independent variables and the cognitive impairment as a dependent variable. In the unadjusted model, IASBPD ≥ 10 mmHg was associated with an increased risk of cognitive impairment (Model 1: *OR* = 1.871; 95% *CI*: 1.223, 2.864; *P* = 0.004). This association remained significant in the subsequent multivariate models, including the fully adjusted model (Model 4: *OR* = 1.902; 95% *CI*: 1.189, 3.040; *P* = 0.007). In addition, there was an 81.3% increased risk of cognitive impairment among subjects with IADBPD ≥ 10 mmHg compared with people with IADBPD < 10 mmHg (Model 1: *OR* = 1.813; 95% *CI*: 1.135, 2.896; *P* = 0.013). However, this association was no longer significant after adjustment for confounders (*P* > 0.05).

**TABLE 4:** Results of multivariate logistic regression of IASBPD and IADBPD with cognitive impairment.

Variables	Cognitive impairment, *OR* (95%*CI*), *P*-value			
	Model 1	Model 2	Model 3	Model 4
**IASBPD**				
<10 mmHg	1	1	1	1
≥10 mmHg	1.871 (1.223, 2.864), **0.004**	1.829 (1.157, 2.892), **0.010**	1.875 (1.200, 2.927), **0.006**	1.902 (1.189, 3.040), **0.007**
**IADBPD**				
<10 mmHg	1	1	1	1
≥10 mmHg	1.813 (1.135, 2.896), **0.013**	1.302 (0.795, 2.132), 0.295	1.591 (0.981, 2.580), 0.060	1.308 (0.791, 2.161), 0.295

Model 1: No adjustment for covariates. Model 2: Demographic characteristics (age, sex, marital status, education) were adjusted. Model 3: Cardiovascular risk factors (smoking, regular exercise, hypertension, CCVD) were adjusted. Model 4: All variables in models 2 and 3 were adjusted. *OR*, odds ratio; *CI*, confidence interval; IASBPD, inter-arm systolic blood pressure difference; IADBPD, inter-arm diastolic blood pressure difference; CCVD, cardio-cerebral vascular disease. Bold values indicate statistically significant differences.

### 3.3 Dose-response relationship between IASBPD and cognitive impairment

**FIGURE 2:**
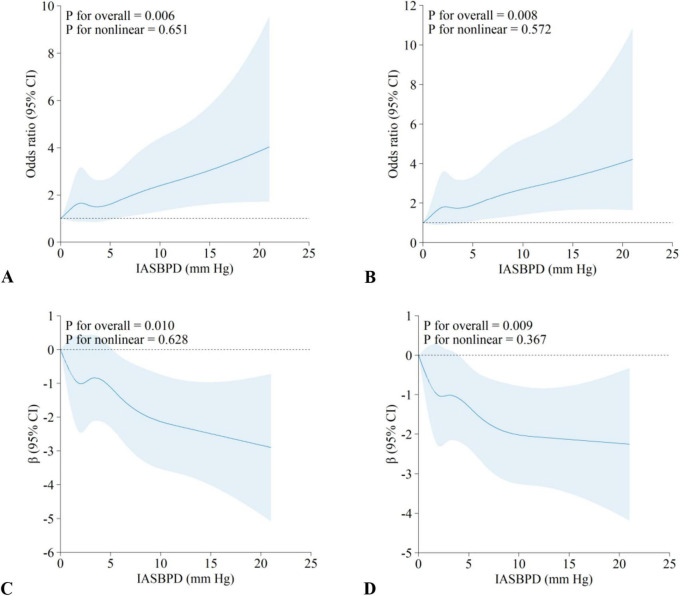
Dose-response relationships between IASBPD and cognitive impairment. Results are from restricted cubic spline regression with nodes at 5th, 27.5th, 50th, 72.5th, 95th. **(A,B)** The association between IASBPD and cognitive impairment; **(C,D)** the association between IASBPD and MMSE scores. **(A,C)** Unadjusted for confounders; **(B,D)** adjusted for confounders such as age, sex, marital status, education, regular exercise, smoking, hypertension, and CCVD. *OR*, odds ratio; β, regression coefficient; *CI*, confidence interval; IASBPD, inter-arm systolic blood pressure difference; CCVD, cardio-cerebral vascular disease. The solid lines represent the *OR*s, and dashed lines represent the 95% *CI*s.

As shown in Figure 2A, there was a dose-response relationship between IASBPD values and the risk of cognitive impairment, which showed a positive linear correlation (*P*_*overall*_ = 0.006, *P*_*nonlinear*_ = 0.651). The linear relationship persisted after further adjustment for potential confounders (*P*_*overall*_ = 0.008, *P*_*nonlinear*_ = 0.572) (Figure 2B). In addition, IASBPD values shows a negative linear correlation with the MMSE score (Figure 2C: *P*_*nonlinear*_ = 0.628; Figure 2D: *P*_*nonlinear*_ = 0.367)

### 3.4 Subgroup analyses

**FIGURE 3:**
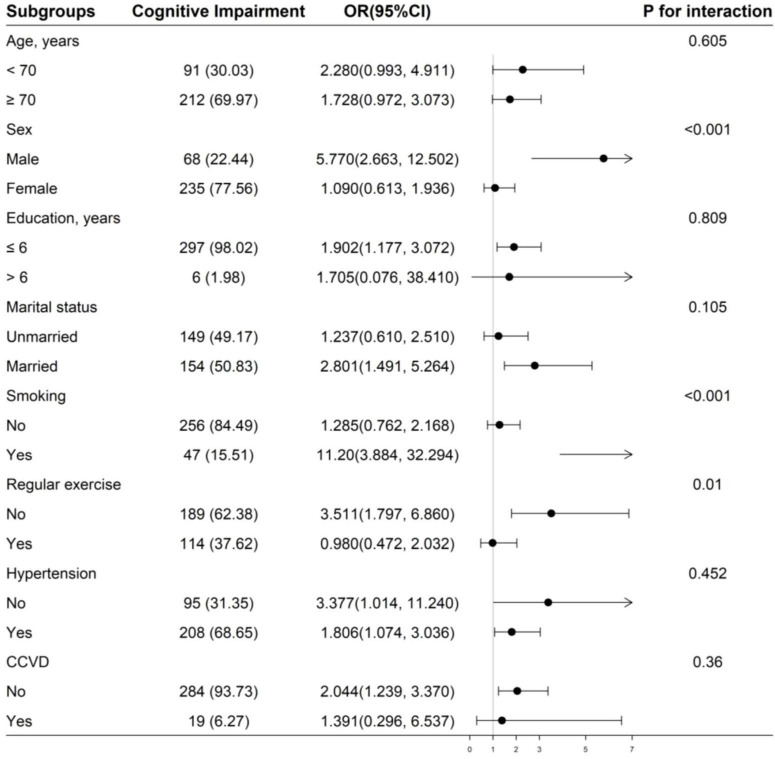
Subgroup analyses of the effect of IASBPD on cognitive impairment. Adjusted for age, sex, marital status, education, smoking, regular exercise, hypertension, and CCVD, if not be stratified. IASBPD, inter-arm systolic blood pressure difference; CCVD, cardio-cerebral vascular disease.

We performed further subgroup analyses to evaluate the effect of IASBPD on cognitive impairment. As shown in Figure 3, the relationship between IASBPD ≥ 10 mmHg and increased risk of cognitive impairment was consistent across the following subgroups: age, education level, marital status, hypertension, chronic kidney disease, and cardiovascular disease (*P* for interaction > 0.05). However, the association between IASBPD ≥ 10 mmHg and increased risk of cognitive impairment stratified by gender, smoking, and regular exercise was significantly different (*P* for interaction < 0.05). In males (*OR* = 5.770; 95% *CI*: 2.663, 12.502), smokers (*OR* = 11.20; 95% *CI*: 3.884, 32.294), and those who lack of regular exercise (*OR* = 3.511; 95% *CI*: 1.797, 6.860), the association between IASBPD ≥ 10 mmHg and increased risk of cognitive impairment was more significant (*P* < 0.05).

## 4 Discussion

In this population-based study, we found that IASBPD ≥ 10 mmHg was independently associated with cognitive impairment in rural Chinese older adults. Specifically, after adjusting for other factors, IASBPD ≥ 10 mmHg was linked to an increased risk of cognitive impairment, with a positive dose-response relationship observed. This association was modified by sex, smoking and regular exercise. Additionally, our results indicated that IADBPD ≥ 10 mmHg was associated with an increased risk of cognitive impairment. However, this association lost significance after adjusting for potential confounders. To our knowledge, this is the first study to explore the association between IABPD and cognitive impairment in rural Chinese older adults. Our results suggested that detecting higher IASBPD may help identify individuals at higher risk for cognitive impairment.

In this study, we defined IABPD as abnormal if the blood pressure difference between the arms of participants exceeded 10 mmHg. According to the guidelines from NICE and Beevers, the range of IABPD below 10 mmHg can be healthy, but more than 10 mmHg may suggest pathology warranting specialist referral (National Clinical Guideline Centre (UK), 2011; Beevers et al., 2001). IABPD ≥ 10 mmHg has been shown to be closely related to early neurological deterioration, cerebral small vessel disease and coronary artery disease (Chang et al., 2018; Chang et al., 2019; Li et al., 2011). Additionally, studies have demonstrated that a threshold of 10 mmHg is the most useful for predicting most outcomes (Pase et al., 2016). Therefore, we recommend that clinicians pay special attention to differences above 10 mmHg when measuring blood pressure in both arms.

To date, there has been limited research on the relationship between IABPD and cognitive function. A cohort study by Clark et al. showed that IASBPD ≥ 5 mmHg was associated with cognitive decline in older adults. When considering the decline in scores on connectivity test, both IABPD ≥ 10 mmHg and IABPD as a continuous variable also showed significant associations (Clark et al., 2020). Another cohort study similarly found that IASBPD ≥ 10 mmHg in older adults was associated with an increased risk of Alzheimer’s disease and subclinical brain injury (Pase et al., 2016). Moreover, a study involving overweight and obese adults with type 2 diabetes found that greater inter-ankle arterial systolic pressure difference was associated with poorer cognitive function (Espeland et al., 2015). Our study results are consistent with previous research, further confirming the relationship between IASBPD ≥ 10 mmHg and an increased risk of cognitive impairment. However, after adjusting for confounding variables, the association between IADBPD ≥ 10 mmHg and cognitive impairment became not significant. This may be related to the different mechanisms by which systolic and diastolic blood pressure affect neurological health (Fuhrmann et al., 2019). Previous literature has found that elevated systolic blood pressure is often associated with arterial stiffness and macrovascular pathologies, which may lead to cognitive impairment by affecting cerebral hemodynamics and vascular function (O’Rourke and Safar, 2005). In contrast, elevated diastolic blood pressure may more frequently reflect abnormalities in peripheral vascular resistance, potentially impairing cognitive function by affecting cerebral microcirculation and blood-brain barrier integrity (Mitchell et al., 2004). Additionally, evidence suggests that in older adults, systolic blood pressure is more closely associated with cardiovascular events than diastolic blood pressure and is a stronger predictor of adverse cardiovascular outcomes (Kershaw et al., 2017). Furthermore, the observed results may be influenced by the study population and methodology.

In our subgroup analysis, we found that the association between IASBPD ≥ 10 mmHg and an increased risk of cognitive impairment was more pronounced in males, smokers, and individuals lacking regular exercise. Among these, smoking and lack of regular exercise have been well documented to be associated with an elevated risk of cognitive impairment (Livingston et al., 2024; Dao et al., 2024). Additionally, these factors may directly or indirectly exacerbate the negative impact of IASBPD on cognitive function in this population by increasing the risk of cardiovascular diseases (Wei et al., 2022). Studies have shown that estrogen plays a significant role in enhancing and protecting cognitive function and is associated with cardiovascular disease protection (Baskaran et al., 2017). Therefore, postmenopausal elderly women are considered a high-risk group for cognitive impairment (Wang et al., 2022). High-risk factors in this population may obscure the true effect of the relationship between IASBPD and cognitive impairment, or the risk conferred by IASBPD may be relatively smaller in high-risk groups, which aligns with the findings of Kim et al. (2019). Furthermore, the study results may be influenced by participant selection, inclusion criteria, and sample size. Therefore, future research should validate these subgroup differences by expanding the sample size to include more diverse populations and employing multiple statistical methods to ensure the robustness and generalizability of the findings.

The exact mechanisms by which IABPD affects cognitive function in elderly individuals remain unclear, but the following hypotheses can be proposed. Firstly, a larger IABPD may indicate the presence of vascular pathologies, such as atherosclerosis and peripheral vascular disease (Lee et al., 2022; Clark et al., 2012), which can lead to vascular stenosis, obstructed blood flow, and subsequently impair cerebral perfusion, thereby exacerbating the risk of cognitive dysfunction (You et al., 2023). Secondly, an IABPD ≥ 10 mm Hg has been shown to be associated with the presence and increased burden of cerebral small vessel disease, suggesting that IABPD may influence cognitive function by affecting cerebral small vessels (Chang et al., 2019; Inoue et al., 2023). Thirdly, a higher IABPD generally reflects an imbalanced state of the vascular system, which may cause vascular endothelial dysfunction or damage, increase cerebrovascular permeability and blood-brain barrier leakage, and contribute to cognitive decline (Hughes et al., 2016). Additionally, numerous studies have demonstrated the association between cardiovascular risk factors and cognitive impairment (Livingston et al., 2024; Austin et al., 2022; Pacholko and Iadecola, 2024). Therefore, the relationship between IABPD and cognitive impairment may be an epiphenomenon driven by similar underlying factors. However, further prospective basic and clinical studies are needed to elucidate the potential mechanisms involved.

International hypertension guidelines recommend that blood pressure should be measured in both arms at the time of diagnosis (Williams et al., 2018). As a simple, rapid, non-invasive, and low-cost indicator, IABPD is suitable for widespread use in primary healthcare institutions. Our findings support the use of IABPD as an effective screening tool for assessing the risk of cognitive impairment in elderly individuals. Therefore, it is recommended to include bilateral blood pressure measurements in routine health check-ups for the elderly, particularly for patients with known cardiovascular risk factors. Additionally, individuals with IABPD ≥ 10 mmHg should undergo further cognitive function assessments to identify potential risks of cognitive impairment early, enabling effective interventions to reduce the incidence of subsequent cognitive decline. Furthermore, targeted health education and behavioral interventions, such as smoking cessation and increased physical activity, may help reduce the occurrence of IABPD, thereby lowering the risk of cognitive impairment (Dao et al., 2024; Yang et al., 2022). However, the predictive and interventional value of IABPD for cognitive impairment still requires further research validation.

The strengths of our study lie in the use of automated and simultaneous measurement technology to assess blood pressure, which provided more accurate IABPD values. Additionally, we adjusted for a large number of potential covariates to account for confounding. However, this study also has several limitations. Firstly, as a cross-sectional study, it can only provide clues to the etiology, and further prospective studies are needed to establish the causal relationship between IABPD and cognitive function, particularly for IADBPD. Secondly, the lack of long-term follow-up limits insight into the temporal relationship between IABPD and cognitive decline. Thirdly, sole reliance on the MMSE might overlook cognitive domains or subtle cognitive deficits. Finally, considering the potential ethnic differences in the association between IABPD and cognition, we should be cautious in generalizing our findings to other racial groups.

## 5 Conclusion

In this study, we found that IASBPD ≥ 10 mmHg is associated with an increased risk of cognitive impairment among elderly individuals in rural Southwest China, and there is a positive dose-response relationship. This association is more pronounced among males, smokers, and those who lacked regular exercise. These findings suggest that IASBPD may serve as useful physiological indicator for identifying individuals at risk of cognitive impairment.

## Data Availability

The raw data supporting the conclusions of this article will be made available by the authors, without undue reservation.
