# Supplementary material for: Large inter-arm systolic blood pressure difference is associated with cognitive impairment in older adults: a cross-sectional study in rural southwest China
Source: Front Aging Neurosci. 2025 Apr 14;17:1489033. doi: 10.3389/fnagi.2025.1489033 (PMC12034631; doi:10.3389/fnagi.2025.1489033)
Supplement: Supplementary file 1 [file Table_1.docx]

**Supplemental Material**

| 6 cities  3 autonomous prefectures  Guizhou Province  12 Villages  3 Villages  3 Villages  3 Villages  3 Villages  Maling Town  Changming Town  Jiuzhi Town  Qingyan Town  Guiyang City  (including 4 districts and 3 counties)  Guiding County  (including 20 towns)  Huaxi District  (including 9 towns)  Qiannan prefecture (including 12 counties) |
| --- |
| **FIGURE S1.** The process of multi-stage cluster sampling. |

| **TABLE S1.** characteristics and comparison of study subjects according to the IASBPD and IADBPD group | | | | | | | |
| --- | --- | --- | --- | --- | --- | --- | --- |
| Variables | IASBPD | | |  | IADBPD | | |
|  | <10mmHg  (n=989) | ≥10mmHg  (n=99) | *P* value | <10mmHg  (n=1008) | | ≥10mmHg  (n=80) | *P* value |
| Age, years |  |  | 0.156 |  | |  | **0.002** |
| > 70 | 443 (44.79) | 37 (37.37) |  | 458 (45.44) | | 22 (27.50) |  |
| ≥ 70 | 546 (55.21) | 62 (62.63) |  | 550 (54.56) | | 58 (72.50) |  |
| Sex |  |  |  |  | |  |  |
| Male | 423 (42.77) | 38 (38.38) | 0.400 | 446 (44.25) | | 15 (18.75) | **< 0.001** |
| Female | 566 (57.23) | 61 (61.62) |  | 562 (55.75) | | 65 (81.25) |  |
| Education, years |  |  |  |  | |  |  |
| ≤ 6 | 868 (87.77) | 87 (87.88) | 0.974 | 882 (87.50) | | 73 (91.25) | 0.324 |
| > 6 | 121 (12.23) | 12 (12.12) |  | 126 (12.50) | | 7 (8.75) |  |
| Marital status |  |  |  |  | |  |  |
| Unmarried | 374 (37.82) | 41 (41.41) | 0.482 | 374 (37.10) | | 41 (51.25) | **0.012** |
| Married | 615 (62.18) | 58 (58.59) |  | 634 (62.90) | | 39 (48.75) |  |
| Smoking |  |  |  |  | |  |  |
| No | 714 (72.19) | 77 (77.78) | 0.234 | 719 (71.33) | | 72 (90.00) | **< 0.001** |
| Yes | 275 (27.81) | 22 (22.22) |  | 289 (28.67) | | 8 (10.00) |  |
| Drinking |  |  |  |  | |  |  |
| No | 720 (72.80) | 78 (78.79) | 0.199 | 728 (72.22) | | 70 (87.50) | **0.003** |
| Yes | 269 (27.20) | 21 (21.21) |  | 280 (27.78) | | 10 (12.50) |  |
| Regular exercise |  |  |  |  | |  |  |
| No | 535 (54.10) | 47 (47.47) | 0.208 | 544 (53.97) | | 38 (47.50) | 0.264 |
| Yes | 454 (45.90) | 52 (52.53) |  | 464 (46.03) | | 42 (52.50) |  |
| BMI, kg/m^2^ |  |  |  |  | |  |  |
| > 25 | 778 (78.67) | 66 (66.67) | **0.006** | 785 (77.88) | | 59 (73.75) | 0.394 |
| ≥ 25 | 211 (21.33) | 33 (33.33) |  | 223 (22.12) | | 21 (26.25) |  |
| Hypertension |  |  |  |  | |  |  |
| No | 387 (39.13) | 17 (17.17) | **< 0.001** | 389 (38.59) | | 15 (18.75) | **< 0.001** |
| Yes | 602 (60.87) | 82 (82.83) |  | 619 (61.41) | | 65 (81.25) |  |
| Dyslipidemia |  |  |  |  | |  |  |
| No | 823 (83.22) | 73 (73.74) | **0.018** | 839 (83.23) | | 57 (71.25) | **0.007** |
| Yes | 166 (16.78) | 26 (26.26) |  | 169 (16.77) | | 23 (28.75) |  |
| Diabetes |  |  |  |  | |  |  |
| No | 924 (93.43) | 95 (96.00) | 0.324 | 943 (93.55) | | 76 (95.00) | 0.609 |
| Yes | 65 (6.57) | 4 (4.00) |  | 65 (6.45) | | 4 (5.00) |  |
| CCVD |  |  |  |  | |  |  |
| No | 904 (91.41) | 82 (82.83) | **0.005** | 917 (90.97) | | 69 (86.25) | 0.163 |
| Yes | 85 (8.59) | 17 (17.17) |  | 91 (9.03) | | 11 (13.75) |  |
| Data are n (%).  BMI, body mass index; CCVD, cardio-cerebral vascular disease. | | | | | | | |
